# Can ventilator settings reduce the negative effects of endotracheal suctioning? Investigations in a mechanical lung model

**DOI:** 10.1186/s12871-016-0196-z

**Published:** 2016-06-27

**Authors:** Espen R. Nakstad, Helge Opdahl, Fridtjof Heyerdahl, Fredrik Borchsenius, Ole H. Skjønsberg

**Affiliations:** 1 Department of Acute Medicine, Oslo University Hospital, Ullevaal, Oslo, Norway; 2 Department of Pulmonary Medicine, Oslo University Hospital, Ullevaal, Oslo, Norway; 3 University of Oslo, Oslo, Norway

**Keywords:** Endotracheal suctioning, Airway pressure, Peak pressure, End-expiratory pressure, Bronchoscopic suctioning, Closed Catheter System, High frequency trigging, Pressure Controlled Ventilation, Volume Controlled Ventilation, AutoPEEP, Ventilator

## Abstract

**Background:**

The insertion of suction devices through endotracheal tubes (ETTs) increases airway resistance and the subsequent suctioning may reduce airway pressures and facilitate atelectasis.

The aim of this study was to investigate how airway pressures and tidal volumes change when different combinations of suction equipment and ETT sizes are used, and to what extent unfavorable effects can be ameliorated by choice of ventilator settings.

**Methods:**

A mechanical ventilator was connected to a lung model by ETTs of 9 mm, 8 mm or 7 mm internal diameter (ID) with a pressure transducer inserted distal to the ETT. The effects of suction procedures with bronchoscope and closed catheter systems were investigated during pressure controlled ventilation (PCV) and volume controlled ventilation (VCV). In each mode, the effects of changes in inspiration:expiration (I:E) ratio, trigger sensitivity and suction pressure were examined.

**Results:**

The variables that contributed most to negative model airway pressures and loss of tidal volume during suctioning were (in descending order); 1) Small-size ETTs (7–8 mm ID) combined with large diameter suction devices (14–16 Fr); 2) inverse I:E ratio ventilation (in VCV); 3) negative ventilator trigger sensitivity; and 4) strong suction pressure. The pressure changes observed distal to the ETTs were not identical to those detected by the ventilator.

**Conclusions:**

Negative model airway pressure was induced by suctioning through small-size ETTs. The most extreme pressure and volume changes were ameliorated when conventional ventilator settings were used, such as PCV mode with short inspiration time and a trigger function sensitive to flow changes.

**Electronic supplementary material:**

The online version of this article (doi:10.1186/s12871-016-0196-z) contains supplementary material, which is available to authorized users.

## Background

Closed catheter systems and flexible bronchoscopes are often used to remove pulmonary secretions from patients with endotracheal tubes (ETTs) or cannulas [1]. As the insertion of a suction device partly obstructs the lumen, air trapping may occur distal to the ETT followed by generation of negative airway pressures during suctioning. Suction procedures may, therefore, induce atelectasis and compromise gas exchange [2]. Other potential hazards include lung collapse [3] and haemodynamic instability [4, 5]. The magnitude of the pressure changes imposed by endotracheal suctioning may not be reliably detected by sensors in the ventilator air circuit [6].

Our main hypotheses were that increased obstruction, as a result of large suction device diameter and/or narrow ETT internal diameter, would contribute to negative model airway pressures during suctioning, and that both pressure and volume changes can be influenced by ventilator settings.

## Methods

### Lung model, ventilator and pressure measurements

**Fig. 1:**
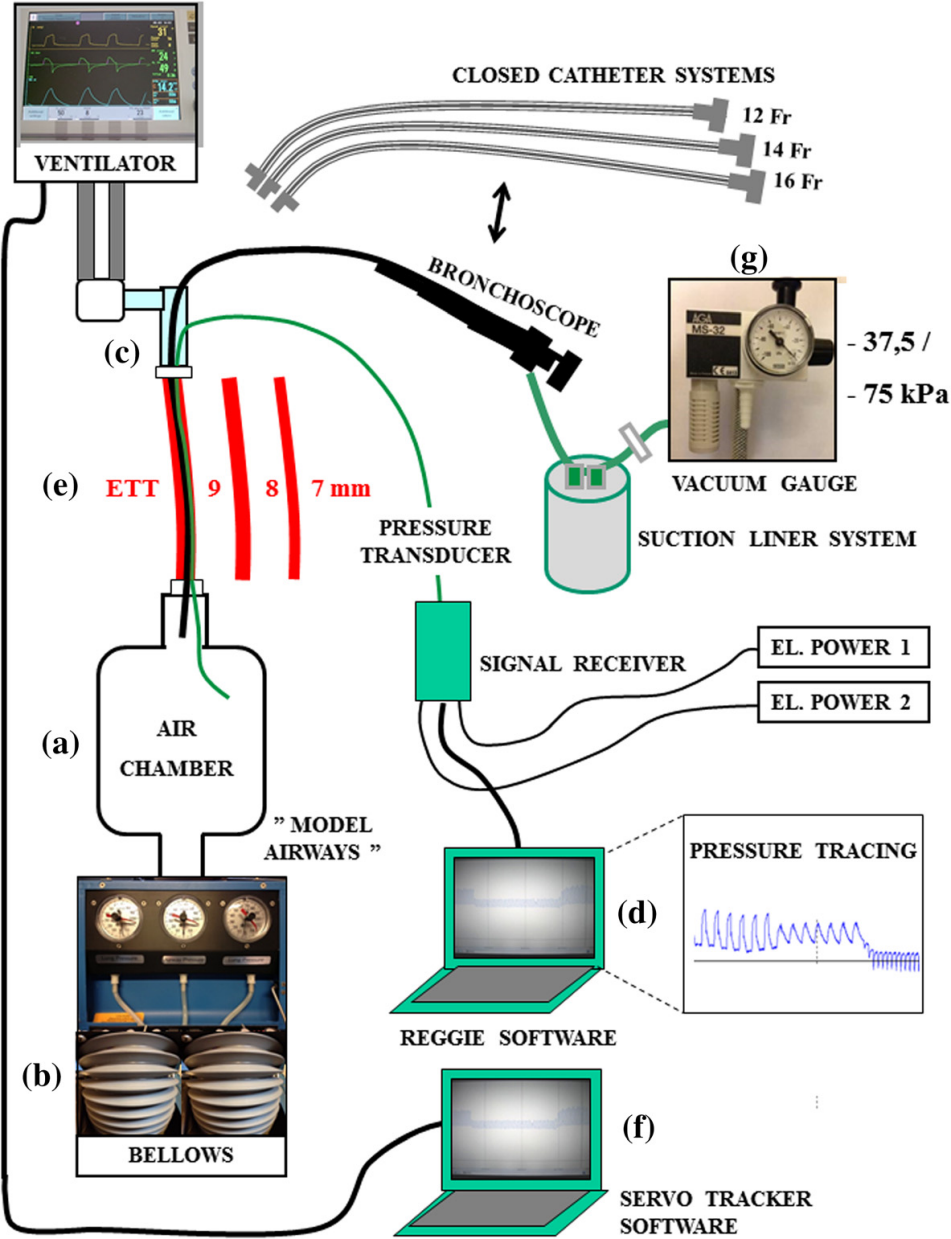
Mechanical lung model test system: Design setup and connections. The term “model airways” (MA) describes the air space distal to the ETT and is comprised of an air chamber, two test model bellows and connecting tubes. Pressures distal to the obstruction were measured by a transducer. Pressures proximal to the obstruction were measured in the ventilator circuit

A commercial test model (Adult/ Pediatric Demonstration Lung Model, IngMar Medical, Pittsburgh, PA, USA) was modified to mimic the low FRC and lung compliance of most ARDS patients and provide means for measuring airspace pressure distal to the ETT during a systematic test protocol. The model was connected to an air chamber (Fig. 1a) to obtain an end-expiratory gas volume of 1400 ml, comparable to the Functional Residual Capacity (FRC) reduction found in mechanically ventilated patients with secondary lung disorder [7]. An extra weight of approximately 750 grams was placed on the bellows to simulate the reduction in compliance common in patients with acute respiratory failure (Fig. 1b).

**Fig. 2:**
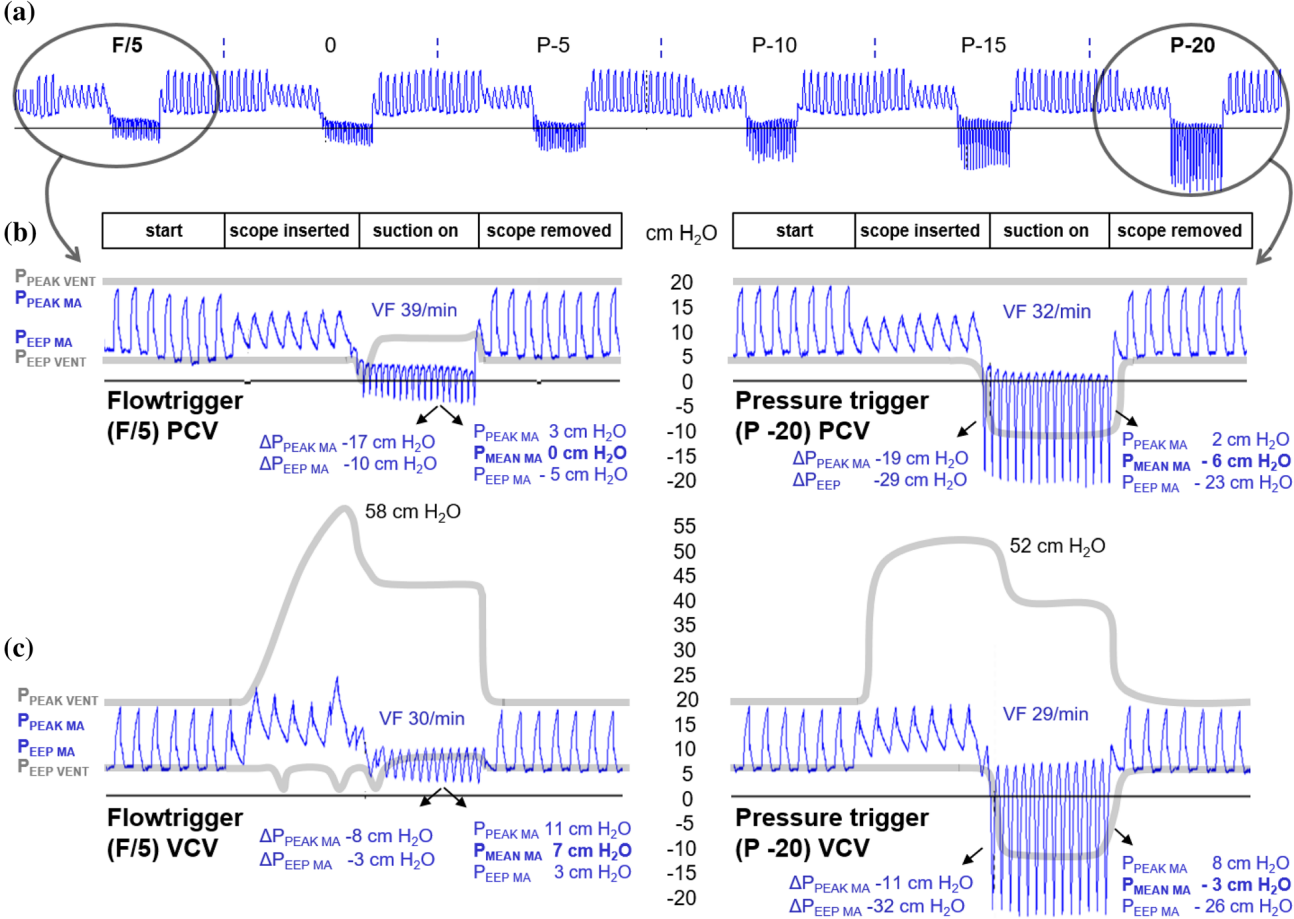
Pressure tracings and ventilator recordings (I:E ratio 1:2, 7 mm ID ETT). **a** Effects of altered trigger sensitivity on peak and end-expiratory model airway pressures (blue tracings, P_PEAK MA_ and P_EEP MA_) are shown during bronchoscope insertion (16 Fr) through a 7 mm ID ETT and with suction pressure −765 cm H_2_O (−75 kPa). Note (both circles) that the pressures, which are measured distal to the obstruction, fall as the bronchoscope is inserted and drop below zero when suctioning is performed. **b** Details of the most and least sensitive triggers with their corresponding ventilator circuit pressures (grey lines; P_PEAK VENT_ and P_EEP VENT_). The total effect of suction compared to normal ventilation (prior to scope insertion) is shown as Δ P_PEAK MA_ and Δ P_EEP MA_. VF = ventilator frequency. Note that ventilator peak pressures, which are measured in the ventilator circuit, are much higher than the actual pressures distal to the obstruction for as long as the bronchoscope is inserted. Ventilator end-expiratory pressures are also much higher than model end-expiratory pressures during suctioning. **c** Details of the same trigger settings during VCV mode, shown as in panel B. Note that for both modes, triggering of new inspirations during suctioning increases the ventilation rate and prevents further reduction of P_EEP MA_

Airspace pressures distal to the ETT (simulating airway pressures distal to an ETT in positive pressure ventilated patients and referred to as “model airways” (MA) in figures) were measured by a rapid response pressure transducer (baud rate 115200/sec) imbedded in a 5 Fr plastic tube (Reggie, Camtech AS, Høvik – Norway) inserted through an air-tight entrance port (Fig. 1c). The transducer was connected to a dedicated computer (Fig. 1d) which displayed and saved real-time pressure changes. Representative tracings from the experiments are depicted in Fig. 2.

During mechanical ventilation, the model was connected to a commonly used ICU ventilator (Servo-i, Maquet, Solna - Sweden) with ETTs of either 9 mm, 8 mm or 7 mm internal diameter (ID) (Mallinckrodt, Hazelwood, Missouri - USA) cut to lengths of 26, 25 and 24 cm, respectively, to simulate clinically relevant ETT lengths in accordance with practise in our hospital (Fig. 1e). Pressures proximal to the ETT (i.e. those detected by transducers monitoring pressures in the ventilator circuit), as well as other ventilator data, were recorded using a commercial filing system (Servo Tracker software version 4.0, Maquet, Solna - Sweden) (Fig. 1f). In addition, a video camera recorded real-time curves and parameters displayed on the ventilator screen.

### Suction devices and flow rates

Bronchoscopic suctioning was performed using a 16 Fr bronchoscope (Olympus LF-TP, Tokyo - Japan) with a suction channel diameter of 2.6 mm. Catheter suctioning was performed using closed system catheters with an outer diameter of 12 Fr in 7 mm ID ETT and 14 Fr in 8 and 9 mm ID ETT, as in clinical practice.

Catheters and bronchoscopes were connected to an AGA MS-32 ejector suction device (AGA, Espoo, Finland) (Fig. 1g) with a vacuum gauge (WIKA Instrument Corporation, Georgia, USA) connected to a suction liner system (Serres Hospital Products, Kauhajoki, Finland). The suction equipment was driven by the hospital compressed air system and generated a negative pressure of −765 cm H_2_O (−75 kPa) (checked against a water column) when set to maximum. Experimental data to support an appropriate maximum level are lacking in the literature. Based on clinical practice, both the maximum level and a moderate level of −382 cm H_2_O (−37.5 kPa) were used in our investigation.

Flow rates through the different suction devices were measured by a spirometer (Vmax 22, Viasys Inc. Yorba Linda, CA, USA). The flow rate through the bronchoscope suction channel was 8.8 l/min at −382 cm H_2_O (−37.5 kPa) and 14.1 l/min at −765 cm H_2_O (−75 kPa). In 12 Fr catheters the flow rate was 9.6 and 15 l/min, respectively, and in 14 Fr catheters 9.6 and 17 l/min. These flow rates correspond with measurements in other experimental studies [3, 8].

### Experimental procedures

The effect of variations in endotracheal tube size (9 mm, 8 mm and 7 mm ID), I:E ratio (1:3, 1:2, 1:1, 2:1, 3:1, 4:1) and flow (F)- or pressure (P) trigger settings (F/5, 0, P-5 cm H_2_O, P-10 cm H_2_O, P-15 cm H_2_O, P-20 cm H_2_O) were investigated with the ventilator set in either pressure control (PCV) or volume control (VCV) mode, using both a bronchoscope and a closed catheter suction system with two suction levels (864 permutations in total). Changes in ventilator tidal volume, circuit pressure and model airway pressure distal to the ETT (P_MA_), were measured for 30 seconds; i) before insertion of a suction catheter/bronchoscope, ii) after insertion but before suctioning, iii) during suctioning with the end of the catheter/bronchoscope positioned 5 cm distal to the ETT, and iv) after removal of the suctioning device; as depicted in Fig. 2.

For experiments with PCV, the ventilator settings were; P_PEAK_ 20 cm H_2_O, PEEP 5 cm H_2_O, ventilator frequency 15/min and RT 5 %. During VCV, initial settings were; inspiratory tidal volume (Vt) 500 ml, PEEP 5 cm H_2_O, ventilator frequency 15/min, pressure rise time (RT) 5 % and pause 0 %, giving a P_PEAK_ of 18–22 cm H_2_O. The model compliance, calculated for both modes, was approximately 39 mL/cm H_2_O. Alarms and cutoff levels for high pressures were set to maximum. For the purpose of this investigation, the level of flow trigging most commonly used in our hospital (bias flow decreased by 1 l/min, called F/5) and five other levels of pressure trigging were used (see above). Changes in ventilator rate and tidal volume were recorded continuously. Mean model airway pressure (P_MEAN MA_) was calculated from the tracings.

### Statistics and data management

Pressures and tidal volumes measured *before* and *after* scope/catheter insertion at each given mode, I:E ratio and ETT dimension (*n* = 12) were analyzed using the statistical package SPSS 15.0 for Windows (SPSS Inc., Chicago, IL, USA). Wilcoxon signed rank test was used to assess possible differences between paired values. Null hypothesis were rejected if two-tailed *p*-value was < 0.05.

Pressures and volumes measured *during* suctioning were strongly influenced by the choice of ventilator trigger sensitivity and suction level (due to high frequency ventilator trigging) and therefore unique for each of the 12 permutations.

## Results

Due to the large number of permutations included in this study, only data illustrating the most salient points are presented. Effects on model airway pressures and ventilation are described with reference to tables and figures. Conventional ventilator setting (PCV with I:E ratio 1:2 and flowtrigger F/5) applies if not otherwise stated.

### A) Effects of suction device insertion

**Fig. 3:**
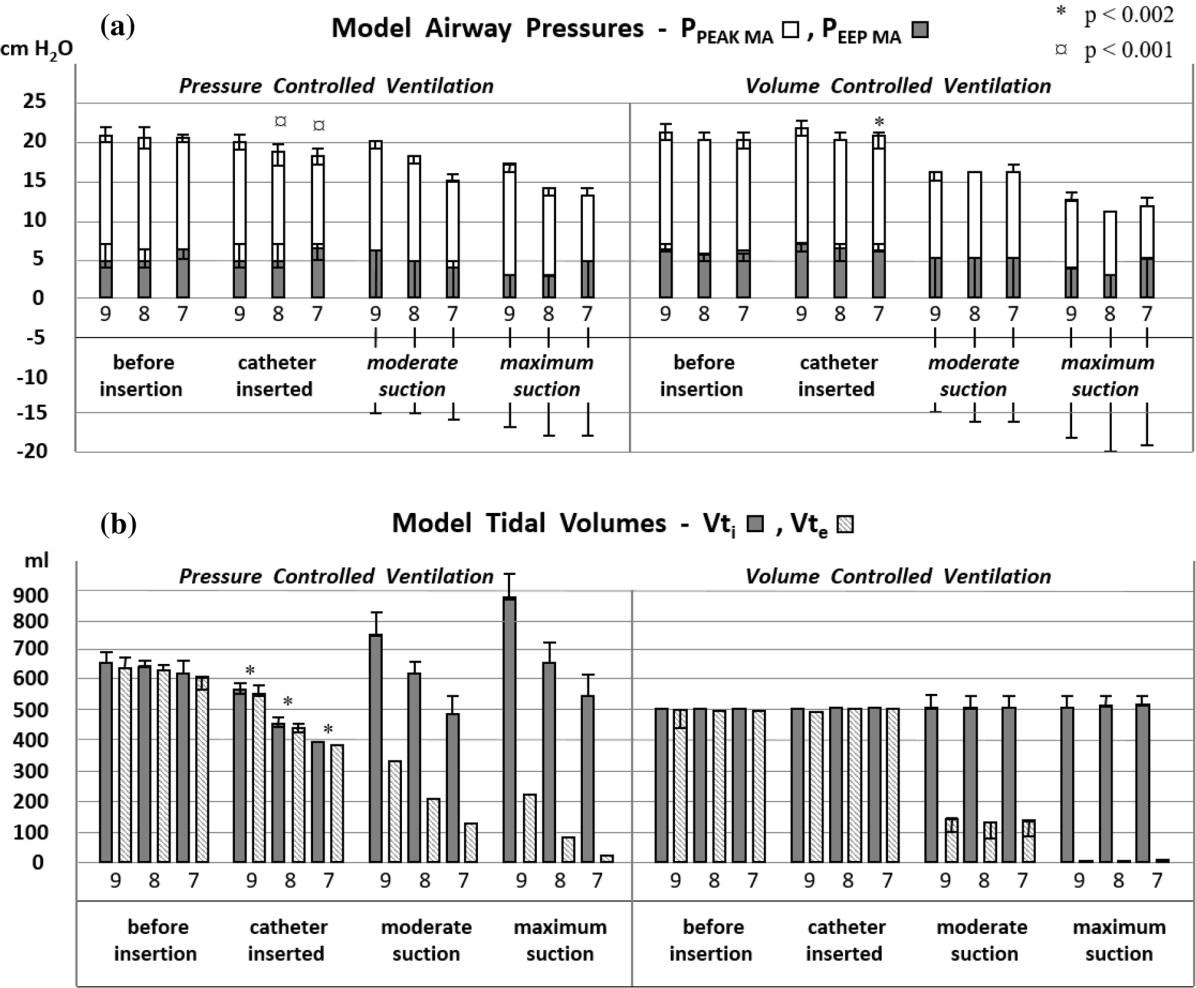
Closed catheter suctioning. Model airway pressures (P_PEAK MA_ and P_EEP MA_, panel **a**) and inspiratory/expiratory tidal volumes (panel **b**) detected by the ventilator (Vt_i_ / Vt_e_) are shown during suctioning with closed catheters (14 Fr in 8 and 9 mm ID ETT and 12 Fr in 7 mm ID ETT). Data are from experiments in PCV (left) and VCV (right) with I:E ratio 1:2 and flowtrigger. Measurements with trigger settings P0, P-5, P-10, P-15 and P-20 cm H_2_O are shown as ranges. The choice of trigger setting and suction level (12 combinations used in this experiment) did not affect measurements *prior* to suctioning; significant changes from catheter insertion are therefore shown accordingly. *During* suctioning, however, pressures and volumes were strongly influenced by high frequency ventilator trigging. The dynamics of such trigging depends on the choice of suction pressure and ventilator trigger sensitivity. Pressures and volumes measured *during* suction were therefore unique for each of the 12 permutations (thus not subject to statistical analysis)

**Fig. 4:**
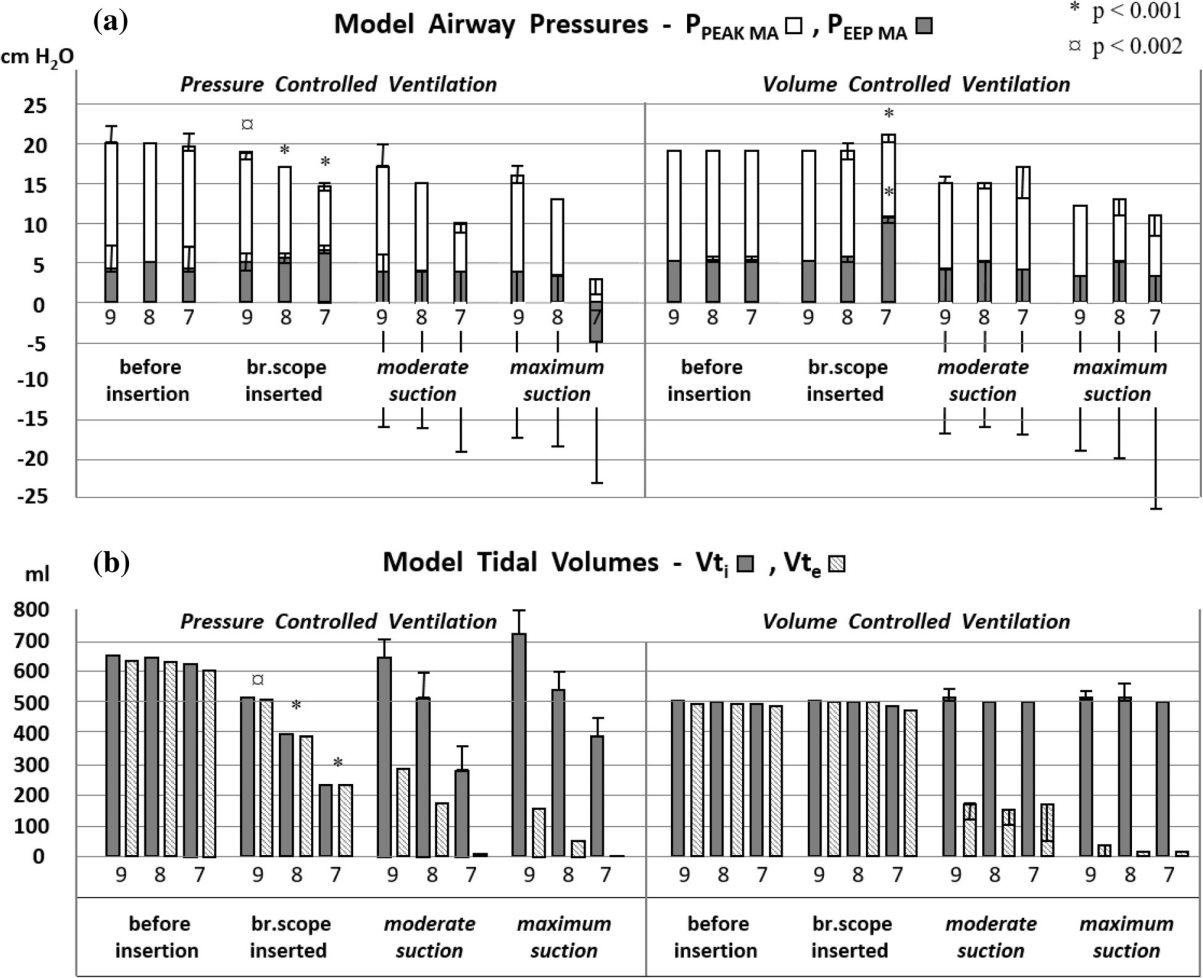
Bronchoscopic suctioning. Bronchoscope insertion and suctioning with altered tube size (ETT 9 mm, 8 mm and 7 mm ID); presented as described in Fig. 3. Bronchoscopic suctioning reduced model airway pressures and tidal volume more than closed catheter suctioning in all ETT sizes, most pronounced in 7 mm ID ETT

The insertion of suction devices in ETTs reduced peak pressures and tidal volumes, as shown in Table 1 (1) and Figs. 3 and 4 (left panels). In VCV, pressures increased after insertion in ETT 7 mm ID but tidal volumes were unchanged (Figs. 3 and 4, right panels).

**Table 1:** Effects of device insertion and suctioning

(1) Closed catheter insertion (no suction)^¤^					
	P_PEAK MA_	P_EEP MA_	Vt_i_	Vt_e_	Vt_i_ - vt_e_
ETT 9 *before*	22	6	679	659	20
*after*	21	6	583	569	14
ETT 8 *before*	22	6	652	635	17
*after*	20	6	470	461	9
ETT 7 *before*	21	6	593	566	27
*after*	18	7	393	376	17
(2) Closed catheter suctioning (moderate vs. maximum suction pressure)^¤^					
	P_PEAK MA_	P_EEP MA_	Vt_i_	Vt_e_	Vt_i_ - Vt_e_
ETT 9 *mod*	20	6	765	331	434
*max*	17	3	873	226	647
ETT 8 *mod*	18	5	623	216	407
*max*	14	3	660	83	577
ETT 7 *mod*	15	4	492	136	356
*max*	13	5	555	21	534
(3) Bronchoscopic suctioning (moderate vs. maximum suction pressure)^¤^					
	P_PEAK MA_	P_EEP MA_	Vt_i_	Vt_e_	Vt_i_ - Vt_e_
ETT 9 *mod*	17	4	647	294	353
*max*	16	4	724	166	558
ETT 8 *mod*	15	4	513	177	336
*max*	13	3	540	52	488
ETT 7 *mod*	10	4	286	8	278
*max*	3	−5	389	0	389
(4) Effect of volume controlled ventilation (closed catheter system, mod. vs. max. suction pressure)					
	P_PEAK MA_	P_EEP MA_	Vt_i_	Vt_e_	Vt_i_ - Vt_e_
ETT 9 *mod*	16	5	503	150	353
*max*	13	4	506	2	504
ETT 8 *mod*	16	5	506	139	367
*max*	11	3	514	0	514
ETT 7 *mod*	16	5	507	144	363
*max*	12	5	517	9	508
(5) Effect of volume controlled ventilation (bronchoscopic suction, mod. vs. max. suction pressure)					
	P_PEAK MA_	P_EEP MA_	Vt_i_	Vt_e_	Vti - Vt_e_
ETT 9 *mod*	15	4	507	185	322
*max*	12	3	507	43	464
ETT 8 *mod*	15	5	498	164	334
*max*	13	5	510	13	497
ETT 7 *mod*	17	4	499	185	314
*max*	11	3	498	17	481

¤ = PCV, l:E 1:2, flowtrigger (F/5). Mod.suction = -382 cm H_2_0 (-37.5 kPa). Max.suction = -765 cm H_2_0 (-75 kPa)

The table exemplifies an experiment were a conventional ventilator setting is used; I:E ratio 1:2 and flowtrigger F/5. Model airway pressures and tidal volumes are presented with variations in ETT size, suction device, suction pressure level and ventilation mode (PCV in sections 1–3, VCV in section 4–5)

### B) Effects of suctioning with different ETT sizes

Suctioning through smaller ETTs reduced model airway pressures and tidal volumes, as exemplified in Table 1 (2). A change from 9 to 7 mm ID ETT reduced P_PEAK MA_ and P_EEP MA_ during closed catheter suctioning by 25 % and 33 % respectively. Inspiratory tidal volume (Vt_i_) decreased 35 %.

### C) Effects of suction device alteration

Bronchoscopic suctioning reduced model airway pressures and tidal volume more than closed catheter suctioning in all ETT sizes, most pronounced in 7 mm ID ETT, as shown in Table 1 (3), were P_PEAK MA_ was reduced 33 % more by bronchoscopic suctioning. Vt_i_ was reduced 42 % more, and Vt_e_ 94 % more, than during closed catheter suctioning.

### D) Effects of increased suction pressure

The reduction in model airway pressure was more pronounced using maximum vs. moderate suction pressure, exemplified in Table 1 (3). The lowest airway pressures were observed with maximum bronchoscopic suction in a 7 mm ID ETT, giving P_PEAK MA_ and P_EEP MA_ of 3 and −5 cm H_2_O, respectively, representing 70 % and 225 % reduction compared to moderate suction. Vt_i_ increased 36 % when changing from moderate to maximum suction presssue (Table 1 (3)). The total effect of maximum suction compared to *normal ventilation* (prior to scope insertion) is shown as Δ P_PEAK MA_ and Δ P_EEP MA_ in Fig. 2.

### E) Effects of ventilator mode change

Changing from pressure to volume controlled ventilation (VCV) had no dramatic impact on model airway pressures and tidal volumes as long as conventional trigger setting and I:E ratio was used, as shown in Table 1 (4 and 5). VCV contributed more to pressure reduction when suctioning was performed in 9 mm ID ETT. PCV contributed more to pressure reduction when suctioning was performed in ETT 7 mm ID. Vt_e_ was reduced more by maximum suction in VCV than in PCV, as shown in Figs. 3 and 4.

### F) Effects of inverse I:E ratio

**Fig. 5:**
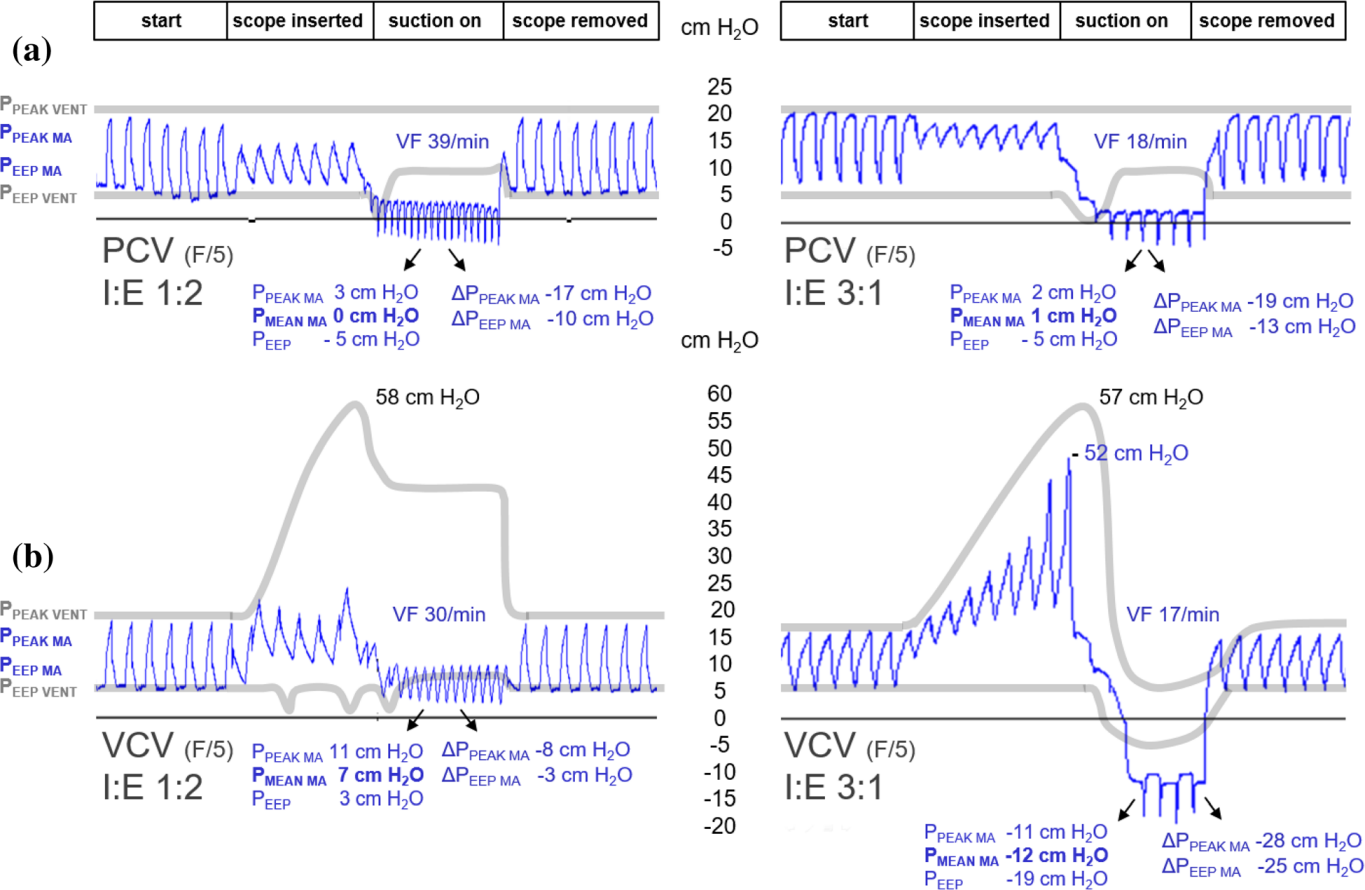
Conventional vs. inverse ratio ventilation. Comparison of conventional (left) and inverse ratio (right) ventilation effects on model airway (P_PEAK MA_, P_EEP MA_) and ventilator circuit (P_PEAK VENT_, P_EEP VENT_) pressures during and after suctioning with maximum suction pressure (−765 cm H_2_O (−75 kPa). A 16 Fr bronchoscope was used in a 7 mm ID ETT with flow trigger (F/5) during PCV (panel **a**) and VCV (panel **b**), presented as in Fig. 2. Note from the blue tracings that the length of the inspiration phase during suctioning is unchanged, while sensitive triggering reduces the expiratory phase substantially. End-expiratory pressures were underestimated by the ventilator during device insertion (P_EEP VENT_ < P_EEP MA_) and overestimated during suction (P_EEP VENT_ > P_EEP MA_)

Inverse ratio ventilation (I:E ratio > 1:1) did not dramatically change model airway pressures during bronchoscopic suctioning in PCV, as exemplified in Fig. 5, upper right panel (I:E 3:1, ETT 7 mm ID). Maximum suction increased Vt_i_ from 234 to 835 ml and reduced Vt_e_ from 216 ml to zero. Much more dramatic effects were seen in VCV, however, as P_PEAK MA_ and P_EEP MA_ escalated immediately after scope insertion and dropped to negative values when maximum suctioning was performed (to −11 cm H_2_O and −19 cm H_2_O respectively), as seen in Fig. 5, lower right panel (I:E 3:1, ETT 7 mm ID). Maximum suction increased Vt_i_ from 496 ml to 871 ml and reduced Vt_e_ from 349 ml to zero.

### G) Effects of altered trigger setting

Setting the ventilator to more negative pressure trigger sensitivity, decreased model airway pressures during bronchoscopic suctioning in both PCV and VCV, as seen in Fig. 2 (right panels). The same applied for closed catheter suction. Tidal volumes, however, were practically unchanged by negative trigger setting.

### H) Pressure escalation effects (autoPEEP)

**Fig. 6:**
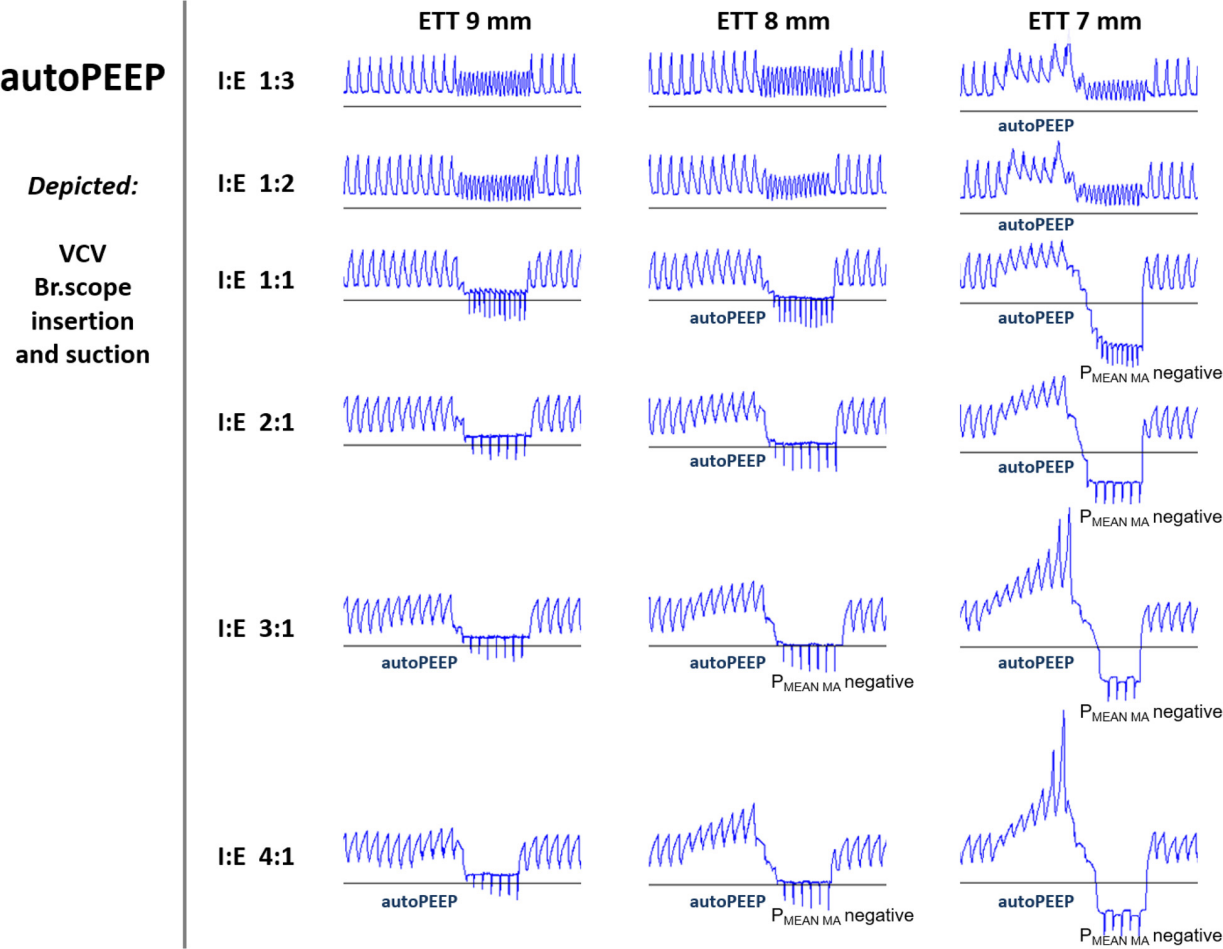
Pressure escalation effects (autoPEEP). Comparison of altered I:E ratios during VCV with three different ETT sizes during insertion of a 16 Fr bronchoscope followed by suction at −765 cm H_2_O (−75 kPa) with flowtrigger (F/5). Presentation of tracings as in Fig. 2, with initial P_PEAK MA_ 20 cm H_2_O and P_EEP MA_ 5 cm H_2_O in all examples. Note that pressures drop to negative values when suctioning is performed with I:E ratio 1:1 and remain unchanged when the ratio is further inversed. Increased end-expiratory pressure (autoPEEP) develops during bronchoscope insertion as a result of progressive air trapping with high resistance and inverse I:E ratio

In VCV, model airway pressures escalated dramatically when a suction device was inserted in a small-size ETT and throughout the next 30 seconds before suctioning was performed. The effect was most profound when a 16 Fr bronchoscope was inserted in a 7 mm ID ETT, as shown in Fig. 6. The volume of air trapped at end expiration escalated with increased inverse ventilation and gave pressure levels of 75 cm H_2_O (P_PEAK_ _MA_) and 25 cm H_2_O (P_EEP_ _MA_) 30 seconds after bronchoscope insertion. Suctioning reduced these pressures within ten seconds to clearly negative values. Pressure escalation effects (autoPEEP) were not seen in PCV.

### I) Ventilator and model airway pressure discrepancies

Pressures measured in the ventilator circuit (shown on the ventilator screen) overestimated the actual model airway pressures distal to the ETT when suction devices were inserted and when suctioning was performed. End-expiratory pressures were underestimated during device insertion (P_EEP VENT_ < P_EEP MA_) and overestimated during suctioning (P_EEP VENT_ > P_EEP MA_), as seen in Figs. 2 and 5 (grey lines).

### J) Effects of high frequency trigging

High frequency trigging was seen *during* suctioning with commonly used I:E ratios, even in 8 and 9 mm ID ETTs. The most sensitive trigger (F/5) increased ventilator frequency from 15 to 39/min during I:E 1:2 (Fig. 2(b)) and up to 50/min in I:E 1:3.

### K) Variables that contribute to pressures reduction and loss of tidal volume

Table 2 summarizes the findings in this bench study with regard to key variables and their impact on reduction of model airway pressures and tidal volumes during ETT suctioning.

An Additional file 1 with more data from this bench test is available in the online version of the article.

**Table 2:** Key variables and their effects on model airway pressure and tidal volume

SUCTION EFFECT VARIABLES	Model airway pressure reduction	Tidal volume reduction
ETT size reduction	Strong impact	Strong impact
Suction device alteration	Bronchoscope > Closed catheter	Bronchoscope > Closed catheter
Increased suction pressure	Strong impact	Vt_e_ reduced
Ventilator PCV - > VCV	Moderate impact (ETT size dependent)	Vt_i_ unchanged, Vt_e_ reduced
Inverse ratio ventilation	Strong impact in VCV (not PCV)	Vt_i_ increased
Negative trigger sensitivity	Strong impact	Minor impact on Vt_i_ and Vt_e_

The variables are listed according to their impact on the reduction of model airway pressure and tidal volume during suctioning

## Discussion

The effects of insertion and use of suction devices distal to ETTs have been examined in prior studies [3, 9]. No one, however, have previously studied to what extent different combinations of suction devices, suctioning pressure levels, and ventilator settings change airway pressures during suction procedures. The use of a lung model for this type of investigation has limitations, the major being that reduced gas volume in patient airways may lead to small airway collapse and therefore a more modest pressure reduction in vivo while increased air trapping could expand the small airways, which would ameliorate pressure increases. Also, the accumulation of secretions in the ETT lumen may reduce its effective inner diameter and enhance negative effects of both device insertion and suctioning in patients [10]. On the other hand, using an artificial lung model makes it possible to systematically record and compare the effects of variations in tube size, suction pressure levels, suction devices and ventilator settings to an extent that would be impossible in patient studies.

### Effects of partial ETT obstruction

The relative proportions between the outer diameter of suction devices and the inner dimensions and lengths of artificial airways determine, to a large extent, the relative obstruction of the ETT lumen and thus the effect on distal airway pressures during suction procedures. Current AARC (American Association for Respiratory Care) clinical practice guidelines suggest that suction catheters should occlude less than 50 % of the lumen of the endotracheal tube in children and adults [1], but there is a considerable discrepancy between guidelines and clinical practice [11]. The guidelines also suggest that suction pressure should be set as low as possible and yet high enough to effectively clear secretions. Commonly used ETT dimensions are 8 to 9 mm ID in males, 7 to 8 mm ID in females and even smaller in youth and children. A bronchoscope has a larger outer diameter but a smaller suction channel than suction catheters; as these factors may interact in determining changes in airway pressure during suctioning procedures, both devices were subject to investigation in this study.

The pressure changes seen in this bench test when ETT dimension was reduced from 8 to 7 mm ID (contrary to shifts from 9 to 8 mm) are to be expected from Poiseuille's Law in which R = 8lŋ/ π r^4^ (R = resistance, l = length of the tube, ŋ = gas viscosity and r = the radius of the tube). Changes to the radius will, by virtue of its elevation to the fourth power, have a strong impact on the resistance; if the tube diameter is reduced to its half, resistance will increase by a factor of sixteen. The equation, however, is not applicable to turbulent flow in which resistance is more profound than in laminar flow. The friction generated by the luminal tube wall and its secretions will also create turbulence, and the insertion of suction devices complicates resistance calculations further. It is clear from the results presented in this study, however, that ETT size does have a major impact on airway pressure and tidal volume, and that the choice of suction device and suction pressure also matters, as summarized in Table 2.

### Effects of ventilator setting and flow

The VCV mode has impact on model airway pressure loss during suctioning. Even if suction is not performed, device insertion in this mode contributes to pressure escalation (autoPEEP). As the ventilator uses the pressure necessary to force a preset tidal volume past the obstruction, a shorter expiration phase (as in inverse ratio ventilation) will reduce the expired volume and increase air trapping.

During PCV, the inspiratory flow decreases as the airway resistance increases, which reduces the tidal volume delivered to distal airways. This may explain why some studies suggest that ETT obstruction influences lung volume loss more than suction pressure in PCV [12]. Even if not tested in our study, compressible gas flow models indicate that no matter the driving pressure, flow will eventually approach (though never fully reach) an upper limit in all constricted tubes.

### Effects of high frequency trigging and inverse I:E ratio

Most ventilators can be configured to deliver a wide range of I:E ratios and allow for triggering of additional inspirations to be initiated either by a reduction in bias flow through the ventilator circuit (flow trigger) or a reduction in circuit pressure (pressure trigger), at the discretion of users. In our model, *high frequency trigging* was seen during suctioning with commonly used ventilator settings, but not when large ETTs (8 or 9 mm ID) were combined with inverse I:E ratio (3:1, 4:1) or negative pressure trigger settings. During *prolonged inspiration* (inverse I:E ratio 1:1–4:1) much of the air volume lost during suctioning was compensated for in PCV due to a high ventilator flow capacity (198 l/min - 3,3 l/sec), which is well above suction flow.

### Clinical considerations

The dramatic pressure changes seen when ETT dimension is reduced from 8 to 7 mm ID, suggests that clinicians should use the largest ETT possible in patients, as a precaution against negative airway pressures when long-lasting suction procedures are performed (as in bronchoscopic suctioning). Larger ETT diameter will also prevent loss of PEEP/recruitment which may otherwise induce atelectasis [2] and other potential hazards [3–5]. Examination of model lungs with different compliances would help to address this issue, and more research is therefore needed.

In a real life ICU setting, removal of pulmonary secretions may be needed several times a day; changing ETT size or suction device in between procedures is therefore not really an option. The parameters that could more easily be changed prior to endotracheal suctioning are ventilator mode, I:E ratio and trigger sensitivity. Unfortunately, changes to these parameters do not seem to counteract unwanted pressure- and volume effects very effectively. On the contrary, it may very well worsen the patient’s condition if wrong choices are made. In our model, positive flow trigger (F/5) and I:E ratio < 1 clearly prevented dramatic changes to airway pressures and ventilation during suctioning. Device insertion contributed to more pressure escalation in VCV compared to PCV. During suctioning, VCV contributed to more pressure reduction when ETT 9 mm ID was used, and PCV contributed more to pressure reduction when ETT 7 mm ID was used.

## Conclusions

The variables that contributed most to negative model airway pressures and loss of tidal volume during suctioning were (in descending order):

1. Small size ETTs (7 mm ID) combined with large diameter suction devices (14–16 Fr).
2. Inverse I:E ratio ventilation (in VCV).
3. Negative ventilator trigger sensitivity.
4. Strong suction pressure.

Conventional ventilator settings ameliorate pressure- and volume changes during both device insertion and suctioning, and seem beneficial also in real life situations.

## Abbreviations

ARDS, acute respiratory distress syndrome; AutoPEEP, increased end-expiratory pressure due to progressive air trapping (as a result of increased expiratory resistance); bpm, beats per minute; ETT, endotracheal tube; Fr, Ch = French (1 Fr = a diameter of 1/3 mm); FRC, functional residual capacity; ICU, intensive care unit; ID, internal diameter; MA, model airways; PCV, pressure controlled ventilation; PEEP, positive end-expiratory pressure; P_EEP MA_, end-expiratory pressure in model airways (distal to ETT); P_PEAK MA_, peak pressure in model airways (distal to ETT); RT, rise time; VCV, volume controlled ventilation; VF, ventilator frequency; Vt_e_, expiratory tidal volume; Vt_i_, inspiratory tidal volume

## Additional file

Additional file 1: RR (Ventilator frequency), Vt_i_ (insp.tidal volume), Vt_e_ (exp. tidal volume), MV_e_ (minute ventilaton), Pinsp (= P_PEAK_ ventilator), Pmean (= P_MEAN_ ventilator), Ventilator PEEP (= P_EEP_ ventilator), Distal transducer Pinsp (= P_PEAK_ model airways), Distal transducer PEEP (= P_EEP_ model airways). Data are presented for each of the permutations listed in the methods section; before scope/catheter insertion (30 sec), after insertion (30 sec), during suctioning (30 sec) and after removal of the device (30 sec). Calculations are also listed in separate columns. (XLSX 522 kb)
